# Interlinked nonlinear subnetworks underlie the formation of robust cellular patterns in *Arabidopsis *epidermis: a dynamic spatial model

**DOI:** 10.1186/1752-0509-2-98

**Published:** 2008-11-17

**Authors:** Mariana Benítez, Carlos Espinosa-Soto, Pablo Padilla-Longoria, Elena R Alvarez-Buylla

**Affiliations:** 1 Instituto de Ecología, Universidad Nacional Autónoma de México, Ciudad Universitaria 3er Circuito Exterior, Junto Jardín Botánico Exterior, Coyoacán 04510, DF, Mexico; 2 Instituto de Investigación en Matemáticas Aplicadas y Sistemas, Universidad Nacional Autónoma de México, DF, Mexico; 3 Centro de Ciencias de la Complejidad, Ciudad Universitaria, DF, Mexico; 4 Department of Biochemistry, University of Zurich, Switzerland

## Abstract

**Background:**

Dynamical models are instrumental for exploring the way information required to generate robust developmental patterns arises from complex interactions among genetic and non-genetic factors. We address this fundamental issue of developmental biology studying the leaf and root epidermis of *Arabidopsis*. We propose an experimentally-grounded model of gene regulatory networks (GRNs) that are coupled by protein diffusion and comprise a meta-GRN implemented on cellularised domains.

**Results:**

Steady states of the meta-GRN model correspond to gene expression profiles typical of hair and non-hair epidermal cells. The simulations also render spatial patterns that match the cellular arrangements observed in root and leaf epidermis. As in actual plants, such patterns are robust in the face of diverse perturbations. We validated the model by checking that it also reproduced the patterns of reported mutants. The meta-GRN model shows that interlinked sub-networks contribute redundantly to the formation of robust hair patterns and permits to advance novel and testable predictions regarding the effect of cell shape, signalling pathways and additional gene interactions affecting spatial cell-patterning.

**Conclusion:**

The spatial meta-GRN model integrates available experimental data and contributes to further understanding of the *Arabidopsis *epidermal system. It also provides a systems biology framework to explore the interplay among sub-networks of a GRN, cell-to-cell communication, cell shape and domain traits, which could help understanding of general aspects of patterning processes. For instance, our model suggests that the information needed for cell fate determination emerges from dynamic processes that depend upon molecular components inside and outside differentiating cells, suggesting that the classical distinction of lineage *versus *positional cell differentiation may be instrumental but rather artificial. It also suggests that interlinkage of nonlinear and redundant sub-networks in larger networks is important for pattern robustness. Pursuing dynamic analyses of larger (genomic) coupled networks is still not possible. A repertoire of well-characterised regulatory modules, like the one presented here, will, however, help to uncover general principles of the patterning-associated networks, as well as the peculiarities that originate diversity.

## Background

Complex interactions among diverse elements underlie the appearance of robust cell patterns during development. Understanding how the topology and dynamics of these interactions are related to phenotypical traits represents one of the most important challenges in systems biology, and is necessary to build a more general theory of development and evolution [1].

Cell-type determination and patterning are key developmental processes. Two overall modes of cell-type determination have been distinguished. The first is the lineage-based mode, which depends on factors transmitted from progenitor to daughter cells. The second one depends on positional information in the context of differentiating cells [2]. The definition of these two types of cell fate determination is instrumental for studying and intervening in developmental systems, especially if one focuses on a particular spatio-temporal interval. The model we present here, however, suggests that the emergence of information needed for cell fate determination results from a dynamic process that depends upon molecular components that are both inside and outside cells. In other words, such information is dynamically generated by the interaction among molecular components within the undetermined cells and those in their context.

Since the generation and maintenance of most cellular patterns may depend on the interplay among gradients, cellular communication, environmental signals and lineage-related mechanisms, the intrinsic *vs. *extrinsic distinction that could be identified with lineage *vs. *positional modes of fate determination is sometimes blurred (see more examples of this claim in [3]). Models that consider gene regulatory networks (GRNs), coupled by cell communication and subject to environmental signals, are thus helpful for an understanding of the emergence of the information needed for cell patterning. Such models will be useful in evaluating to what extent positional or lineage-related mechanisms are distinguishable, independent of undetermined cells, and necessary or sufficient for cell determination and patterning in a particular system.

Dynamical GRN models have been fruitfully used to study cell-fate determination [4-7]. In such models, the steady gene activation states (attractors) to which the dynamic system converges correspond to the multigene configurations that characterise different cell types [8]. A discrete approach to modelling the dynamics of such genetic networks was first introduced by Kauffman [8] in order to describe qualitatively the concerted action of several genes during cell differentiation. This approach has been proven to capture the logic of regulatory interactions key to cell-type specification in several biological systems (e.g. [5-9]). Nevertheless, data availability has limited most GRN models to intracellular behaviour, and therefore cellular communication and other important features of pattern formation have been omitted from most models. On the other hand, mesoscopic models that have considered the latter features [10-12] do not usually incorporate complex intracellular networks that regulate cell-type determination.

The epidermis of the model plant *Arabidopsis thaliana *is one of the most thoroughly studied cell-type determination and patterning systems. Such experimental efforts have enabled the postulation of GRNs for cell-fate determination in this system (see recent reviews in [13-15]). Moreover, relatively simple models may capture relevant aspects of this system as it may be accurately represented in a two-dimensional domain, and because *Arabidopsis *epidermal cells attain their fate in a fixed domain before they elongate. Actually, some theoretical studies on this system have been published [16-20]. Of these studies, only one [20] has considered the spatio-temporal dynamics of cell-fate determination by explicitly modelling GRNs, yet it focused on leaf patterning and was mainly aimed at presenting a useful modelling platform rather than studying a particular patterning mechanism.

We put forward a spatio-temporal model of coupled GRNs that integrates updated experimental evidence for cell-fate determination in *Arabidopsis *epidermis and incorporates cellular communication, domain characteristics (size and boundary conditions) and cell shape. We intend to provide a better understanding of how the dynamics of genetic interactions within a cell, in conjunction with cell-to-cell communication, give rise to robust spatial patterns of gene expression and, concomitantly, to cell type determination and arrangement.

**Figure 1:**
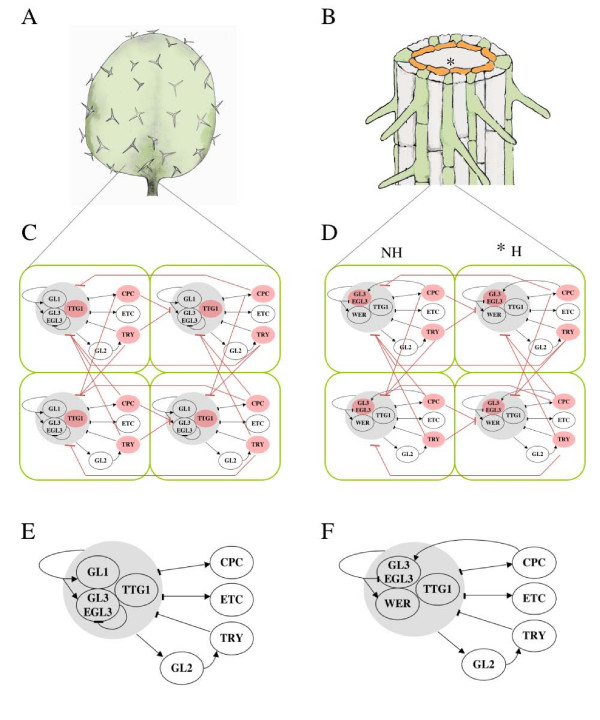
**Cellular patterns and meta-gene regulatory network models for leaf and root *Arabidopsis *epidermis**. Spaced-out pattern of trichome distribution in the leaf of *Arabidopsis thaliana *(A). Root-hairs (green) are arranged in bands that overlie the junction of two cortex cells (yellow) (B). Coupled gene regulatory network (GRN) model for cell type determination in leaf epidermis (C). GRN underlying cell-fate determination in root epidermis (D). (E) and (F) represent the GRN for leaf and root epidermis, respectively. In both networks, nodes correspond to genes, arrows stand for positive regulatory interactions and flat-end edges stand for negative ones. Red nodes represent elements that are able to move among cells and couple the GRN into meta-GRN. Red lines stand for intercellular interactions established by mobile elements. Asterisks in (B) and (D) indicate the H position where the cortex-related signal is acting.

*Arabidopsis *leaf epidermis bears hairs (trichomes) that tend to appear away from each other (Figure 1A; [21]), while root epidermis exhibits trichoblast (root hair precursors) and atrichoblasts arranged in bands of a single cell type (Figure 1B; [22,23]). Despite the contrasting spatial patterns, epidermal cell type determination in root and leaf appears to be associated with similar GRNs (Figures 1E and 1F). Furthermore, although root and leaf GRNs exhibit some differences, they seem to be qualitatively equivalent in dynamical terms [18,24].

The GRNs associated with epidermal cell type determination have been thought to behave as a so-called activator-inhibitor system [25,26] and some theoretical work has been done in this direction [17-19]. In a previous paper, we simplified these GRNs to an activator-inhibitor system, so that we could explore the role of cell contextual or positional traits in cellular spatial patterns [18]. It is often the case, however, that the structure of the GRN confers important dynamic properties on the system and that these properties are not recovered by simplified versions of the system [27]. For example, coupled GRNs in cellularised spatial domains do not necessarily recover the attractors of single-cell GRNs. Moreover, some recent results indicate that the processes giving rise to the epidermal patterns may be richer than an activator-inhibitor system, as different nonlinear sub-networks of the GRN may generate or reinforce such patterns (e.g. [28]). A meta-GRN model that encompasses these sub-networks is required to explore whether coupled GRNs in cellularised spatial domains recover the expected and robust gene activation configurations, and to study the role of sub-network redundancy in pattern formation.

Here we have modelled the meta-GRN by integrating the updated experimental data (Figures 1C–1F) into models that explicitly consider GRNs coupled via cell-to-cell communication in a cellularised domain. Interestingly, the attractors for the single cell GRN [18] were qualitatively preserved in the meta-GRN and the simulated spatial patterns of steady states corresponding to hair and non-hair cells matched those observed in actual *Arabidopsis *wild-type plants. The proposed model therefore accounts for the formation of cellular patterns from initially homogeneous domains containing cells with the same GRN. The model presented here is also useful for exploring the stability of such patterns in the face of environmental and developmental perturbations.

We validated the model by simulating mutant networks and comparing our results with reported gene expression profiles of mutant phenotypes. Then we used the model to postulate novel and testable predictions regarding additional regulatory interactions, the effect of cell shape on patterning and the link between the simulated GRN and the Gibberelic acid signalling pathway. Our results suggest that the proposed GRN constitutes the core of a complex regulatory module associated with *Arabidopsis *epidermal cell type-determination and patterning. Finally, this study contributes to uncovering both generic and specific aspects of GRN coupling mechanisms and patterning processes in biological systems.

## Methods

The gene regulatory network model we postulate here is grounded on available experimental data up to June 2008. This evidence is summarised in the following paragraphs. In both root and leaf epidermis, TRANSPARENT TESTA GLABRA 1 (TTG1), bHLH proteins, GLABRA3 (GL3) and ENHANCER OF GLABRA 3 (EGL3), and Myb proteins, GLABRA1 (GL1) and WEREWOLF (WER) in leaf and root, respectively, form a complex that positively regulates the transcription of *GLABRA2 *(*GL2*). In turn, the expression of *GL2 *determines leaf trichome cell-fate and root atrichoblast identity. *TRIPTYCHON *(*TRY*), *CAPRICE *(*CPC*) and *ENHANCER OF TRIPTYCHON AND CAPRICE 1, 2 and 3 *(*ETC1*, 2 and *3*) repress the leaf and root activators in partially redundant ways. All of these inhibitors are upregulated, directly or indirectly, by the root and leaf activators. Interestingly, *GL2 *positively regulates *TRY *in both root and leaf epidermis [29,30].

*WER *and *GL1 *are assumed to be directly or indirectly upregulated by themselves. *TTG1 *is expressed in all the epidermal cells during hair and non-hair determination and the TTG1 protein moves through plasmodesmata among neighbouring cells in the leaf epidermis, accumulating in trichomes [29,30]. The fact that TTG1 moves among leaf epidermal cells and binds GL3 in the formation of a protein complex has recently led Bouyer and collaborators [28] to postulate a *TTG1*-trapping/depletion mechanism that could explain the accumulation of TTG1 in cells with GL3 (trichomes) and could suffice for trichome pattern formation. This mechanism is discussed later in the context of other experimental data and our results.

Despite being part of the activator complexes, *GL3 *and *EGL3 *are primarily expressed in root hair cells and their proteins move towards cells in which the other components of the complex are present. *GL3 *and *EGL3 *also seem to be activated by the inhibitor *CPC *and repressed by the root activator complex [31,32]. In leaves, *GL3 *expression occurs in trichome cells, where *GL1 *maximum expression is reported. Transcription of *GL3 *seems to be downregulated by itself and the localisation of the GL3 protein within the cell nucleus is regulated by *TTG1 *and *GL1 *[31]. Experimental evidence also shows that *TRY *acts non-cell autonomously in leaves and that the CPC protein moves through plasmodesmata among cells in both leaf and root epidermis [33,34]. In the root, *SCRAMBLED *(*SCM*) seems to be a crucial component of a positional signal downregulating *WER *in root-hair bands and therefore biasing the cellular pattern [35,36].

In the proposed GRN model, elements or nodes of the network correspond to genes. The *ETC *node summarises the partially redundant activity of the three enhancers of *TRY *and *CPC*. Edges of the network stand for regulatory interactions between nodes (activation or repression; Figures 1E and 1F). Nodes representing genes for which there is enough information can attain three different states (0, no expression; 1, mild expression; 2, strong expression). In the leaf system, only *ETC *can either be expressed, 1, or not expressed, 0, and only *ETC *and *TRY *are binary in the root system. For simplicity, the state of all nodes was updated synchronically. In single-cell GRNs, the updating scheme does not seem to affect attractors when these are fixed-point type [37]. Since the attractors of our GRN model are indeed fixed-point ones, it could be the case that asynchronicity does not affect them, yet this issue has not been explored in coupled GRN models.

In the GRN model, each node's state depends on that of other nodes, its regulators. The level of expression of a given gene is represented by a discrete variable *g *(0, 1 or 2) and it depends on the level of expression of other components of the network, *g*_1_, *g*_2_, ..., *g*_*N*_. The state of every gene *g *therefore changes according to:

(1) *g*_*n*_(*t*+1) = *F*_*n*_(*g*_*n*1_(*t*), *g*_*n*2_(*t*), ..., *g*_*nk*_(*t*)).

In this equation *g*_*n*1_, *g*_*n*2_, ..., *g*_*nk *_are the regulators of gene *g*_*n*_, and *F*_*n *_is a discrete function known as logical rule (logical rules were grounded on available experimental data and are graphically represented in Figures 1E, 1F, [see Additional file 1]). Given the logical rules, it is possible to follow the dynamics of the network for any initial configuration of the nodes expression states. Since the GRN is simulated as discrete, all possible combinations of gene activation states may be explored. One of the most important dynamical traits of a GRN is the existence of attractors. Starting out from an initial state, the reiterated application of equation (1) generates dynamics in which genes go through transient states until the whole network enters into a stationary or periodic profile of multi-gene expression. Such stationary or periodic expression profiles constitute the GRNs' attractors, which correspond to gene expression profiles characteristic of particular cell types.

In order to couple the GRNs in a compartmentalised (cellular) domain, we considered a discrete lattice of *n *× *n *elements, in which each element (*i*, *j*) represents a cell with a GRN. As in real organisms, all cells bear the same GRN. Modelled lattices are a simplified representation of leaf or root epidermal sections. Cells in the lattice have exactly four neighbours and there is no difference in permeability between them.

**Figure 2:**
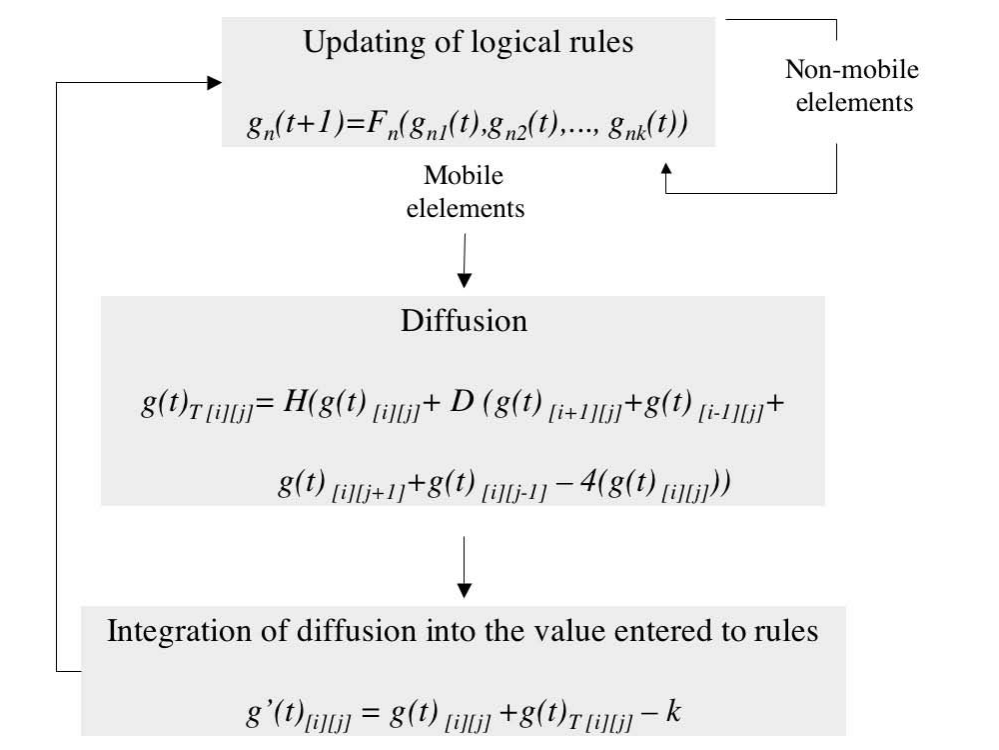
**Diagrammatic representation of the model structure.** In every time-step, the nodes' states are updated according to logical rules [see Additional file 1], then the mobile elements are allowed to diffuse and, finally, diffusion is considered to recalculate the states of mobile elements. These new values are entered into the logical rules in the next iteration. The state of non-mobile elements is only determined by the logical rules applied every time-step.

According to experimental data, some proteins codified by elements of the network move to neighbouring cells and affect gene expression in a non-cell-autonomous fashion (TRY, CPC and TTG1 in the leaf epidermis and CPC, GL3 and EGL3 in the root epidermis), giving rise to a network of coupled networks (herein *meta-GRN*). Although empirical evidence supporting cell-to-cell motion rather than aplopastic transport only exists for CPC and TTG1, all available data are congruent with the assumption that all mobile elements of the GRN move in a cell-to-cell manner. In the spatial model we therefore allowed for certain elements to move among neighbouring cells (Figures 1C, 1D and 2) following the equation:

(2) *g*(*t*)_*T *[*i*] [*j*] _= *H*(*g*(*t*)_[*i*] [*j*]_+ *D*(*g*(*t*)_[*i*+1] [*j*]_+*g*(*t*)_[*i*-1] [*j*] _+ *g*(*t*)_[*i*] [*j*+1]_+*g*(*t*)_[*i*] [*j*-1]_- 4(*g*(*t*)_[*i*] [*j*]_)),

where *g*(*t*)_*T *[*i*] [*j*] _is the total amount of protein *g *in cell (*i*, *j*). *D *is a continuous variable that determines the proportion of *g *that can move from any cell to neighbouring ones and is correlated to the diffusion rate of *g*. *H *is a step function that converts the continuous values corresponding to the amount of *g *diffused into each cell into a discrete variable that may attain values of 0, 1 or 2. In order to simulate the effect on diffusion of protein attachment to a protein complex, our simulations consider that the mobile elements of the network diffuse with a lower rate from inside to outside when the other protein complex components are inside the same cell. So, for instance, when there is GL3 in a cell, the term -4(*g*(*t*)_[*i*] [*j*-1]_) in the diffusion equation of TTG1 is removed or decreased (see also [28]). More generally, we defined a term -*A*(*g*(*t*)_[*i*] [*j*]_), in which de value of *A *depends on the presence of trapping proteins in a cell (*i*, *j*) at time *t *(*A *= 4 when no trapping proteins are present and *A *< 4 when these are present).

*D *was systematically varied from 0.0 to 0.25 with a step size of 0.01, enabling the generation of parameter spaces in which we plotted the average number of ectopic trichoblasts and atrichoblasts for every combination of parameter values. Four types of 'trapping' diffusion terms were also tested (-*A*(*g*(*t*)_[*i*] [*j*]_), with *A *= 0,1,2,3; see Results). In the leaf system, boundary conditions were simulated as zero-flux, while in the root system, two borders were identified and two were kept at zero-flux, simulating a root-like cylinder. In the root model, the positional signal associated with *SCM *was simulated as a constant downregulation of *WER *every three cell files (two NH files between H files) [36].

Then, *g*(*t*)_*T *[*i*] [*j*] _was considered in order to estimate the *g*(*t*)_[*i*] [*j*] _value, which was then used to update the logical rules, according to the equation below:

(3) *g'*(*t*)_[*i*] [*j*] _= *g*(*t*)_[*i*] [*j*] _+*g*(*t*)_*T *[*i*] [*j*] _- *k*,

where *k *stands as a degradation constant. *g'*(*t*) was taken as an input to evaluate the logical rules and obtain *g*(*t*+1)_[*i*] [*j*]_.

In brief, each cell's GRN is initialised with a random gene activation profile. Then, three steps are sequentially repeated until the whole lattice reaches a steady state (diagrammatic representation in Figure 2): (1) application of logical rules, (2) diffusion of mobile elements and (3) integration of diffused protein into the GRN inputs for the next updating step according to the logical rules.

In order to test the model, we also simulated networks that correspond to reported mutants. The loss and gain of function simulations were done by fixing the expression value of the altered gene to *0 *or *2*, respectively. The random seed was the same for both wild-type and mutant simulations so that results were comparable.

It should be noted that there are several dozens of genes involved in hair determination, patterning and differentiation in *Arabidopsis *[38]. We have, however, incorporated in the GRN models only the elements that seem to be responsible for the decision: hair *vs. *non-hair. Changes in expression states of these elements do indeed yield changes on epidermal cell identity. In contrast, most of the genes that were not included seem to act downstream of the GRNs modelled here and may play important roles in morphogenesis once cells are committed to a particular fate. A few other genes that are not considered seem to act upstream of the GRN postulated here, probably linking the GRNs with signalling pathways or tissue-specific factors. Our simulations of both wild-type and mutant networks indeed suggest that the GRNs modelled here incorporate the necessary components to render the robust gene activation profiles characteristic of epidermal hair and non-hair cells in both roots and leaves, and thus uncover the core of a regulatory module that regulates epidermal cell patterning in *Arabidopsis*.

Graphics were elaborated in MATLAB and programs in C (codes available upon request).

## Results

### Gene expression profiles and wild-type spatial patterns are robustly recovered by the leaf GRN model

The spatial model of the leaf system (Figure 1C) was initialised by random assignment of activation states (0, 1 or 2) to the nodes in the GRNs. In this case, TRY, CPC and TTG1 were allowed to move among cells according to experimental data (see previous section). After a few iterations, the GRNs in all cells in the lattice reached one of two attractors. One of these attractors exhibits a GRN activation profile that matches the characteristic profile of trichome precursor cells (black cells in Figure 2A), in which the expression of all network elements peaks. The other attractor matches the gene activation profile reported for pavement or non-trichome cells, in which the networks' elements are less expressed than in trichomes or not expressed at all (white cells in Figure 2A). This result indicates that the GRN structure, interaction rules and protein diffusion functions considered in the leaf GRN model are sufficient to generate the two, and no more, stable expression profiles that mimic those reported for leaf hair and non-hair precursor cells in *Arabidopsis*.

Simulated spatial domains show a dotted pattern similar to that of trichomes in actual leaves. Moreover, the simulated trichome distribution was also significantly more spaced out than expected in a random spatial distribution, as is the case in actual leaves. This was found by calculation of the *R*-value ([39,21]: *R *= 1 in a random distribution; *R *> 1 in a pattern with elements more spaced out than in a random case; and *R *< 1 in a clustered distribution). *Arabidopsis *trichomes of the Col ecotype have an *R *= 1.40 and the clustering probability (*C*) is around 0.08 [21], while the typical values we found in our simulations are 1.2 <*R *< 1.40 and *C *< 0.02 (Table 1). Since our model does not consider growth or proliferation, it is important to note that the *R*-values estimated in real leaves were measured before growth and proliferation affected trichome distribution [21] and are overall comparable to those obtained in our simulations.

**Table 1:** The leaf meta-GRN recovers the spaced-out pattern characteristic of leaf trichomes.

	<R>	<C>
Random	1	0.5
Clustered	< 1	1
Spaced-out	> 1	0
WT leaf	1.4	0.08
Simulation	1.3	0

Measures for matrices with different 'hair' arrangements are presented. *R *indicates how clustered or spaced-out the arrangement is [21], while *C *indicates the clustering probability. Simulations of the leaf meta-GRN model were carried out with *D*_*CPC *_= 0.08, *D*_*TRR *_= 0.08, *D*_*TTG *_= 0.06.

We then asked if the simulated pattern depended on gene interaction details, particular domain characteristics (boundary conditions and size), or specific diffusion parameters, or if it depended on the overall coupled GRNs system. We found that the simulated leaf pattern was robust to changes in boundary conditions. Diffusion parameters *D*_*TRY*_, *D*_*CPC *_and *D*_*TTG*1 _were also systematically varied and the simulated trichome pattern (*R *> 1, *C *≈ 0) was not altered significantly for a wide range of parameter values. The pattern was maintained for 0.01 <*D*_*TRY*/*CPC *_< 0.26, independently of the *D*_*TTG*1 _value. Interestingly, this result indicates that, although the *TTG1*-trapping/depletion mechanism suffices to generate a trichome-like pattern [28], in the context of the whole GRN it may not be a necessary mechanism. This is also suggested by experiments showing that plants overexpressing *GL3 *are able to recover a wild-type phenotype in a *ttg1 *mutant background [40]. The interplay among different potential patterning sub-networks is discussed in more detail below. As mentioned above, diffusion of mobile proteins from a cell [*i*] [*j*] to neighbouring cells was restrained when other interacting proteins were present in the [*i*] [*j*] cell. This was done by taking the term -4(*g*(*t*)_[*i*] [*j*]_) in equation (1) as *A*(*g*(*t*)_[*i*] [*j*]_) with *A *< 4. We varied such a term by taking *A *= 0, 1, ..., 4 and no differences in the simulated wild-type patterns were observed.

Interestingly, the spaced-out pattern was also found to be robust to point alterations of the gene interaction functions. We tested the latter by modifying the output of all logical rules, one at a time. We found that 88% of the alterations yielded the same attractors and trichome-like spatial pattern as the original model. Together these results suggest that the overall GRN topology and the coupling mechanisms, rather than detailed aspects of the network kinetics or specific parameter values, underlie the emergence and stability of the cell patterns in *Arabidopsis *leaf epidermis.

### The root GRN model robustly recovers the profiles and patterns characteristic of the wild-type root epidermis

**Figure 3:**
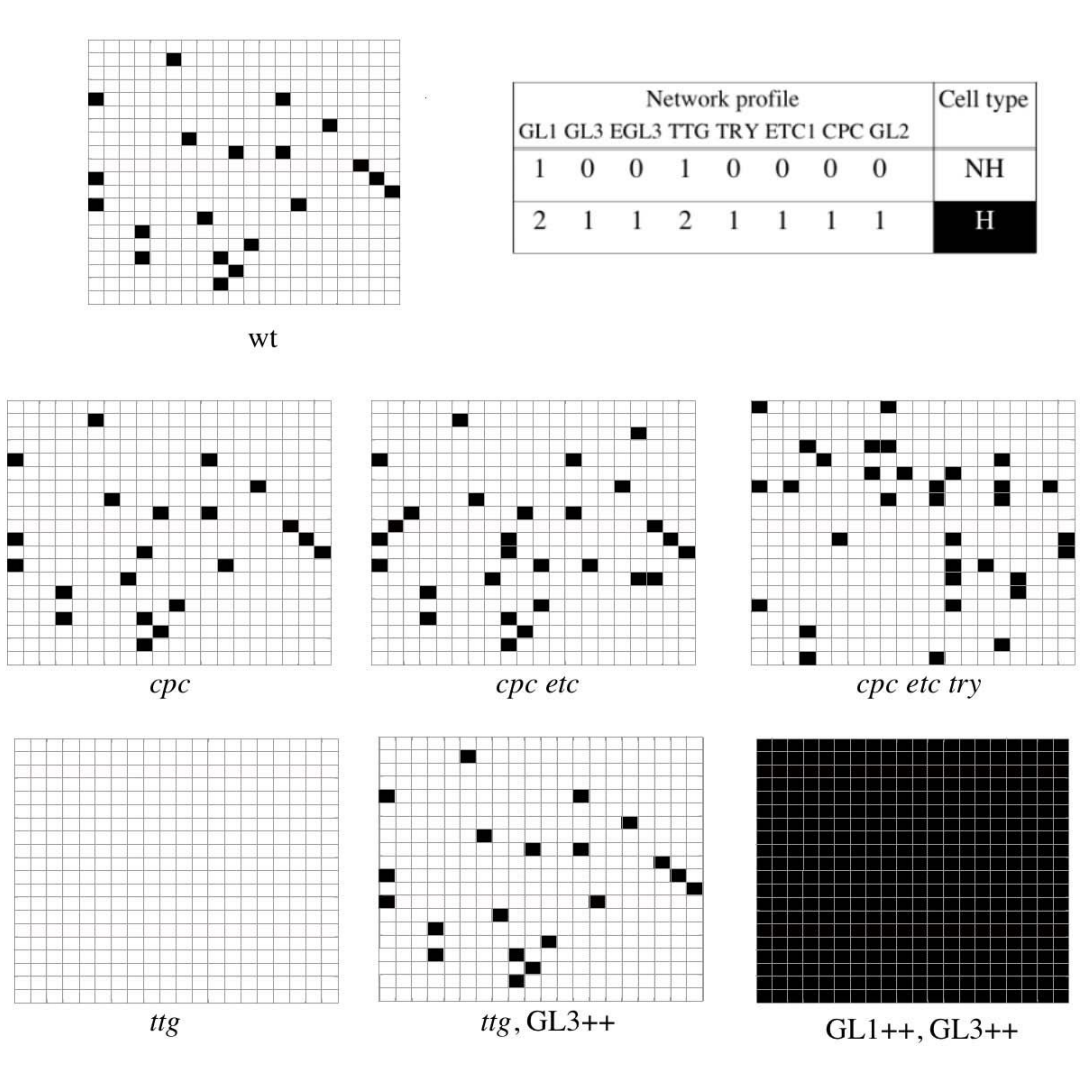
**The model renders cellular patterns similar to those observed in the leaf epidermis**. The simulated cellular patterns for wild-type (wt) and mutant networks are consistent with the patterns reported in the literature. Black squares correspond to trichomes and white ones to pavement cells (non-hair cells). Captions under the matrixes indicate the simulated genotype that gave rise to each of them (++ stands for overexpression, while lower case italics indicate loss of function). The table shows that the network profiles typical of hair and non-hair cells are recovered by the meta-GRN model. These simulations were all performed in 20 × 20 matrices with parameter values *D*_*CPC *_= 0.05, *D*_*TRR *_= 0.05, *D*_*TTG *_= 0.03.

In the root meta-GRN model, CPC and bHLH proteins (GL3 and EGL3) were allowed to diffuse, as suggested by experimental data (see Methods). In this case, the cortical signal associated with *SCM *was simulated every two cell files as a downregulating input on *WER*. GRNs were also randomly initialised and two network attractors were found. In the first (white cells in Figure 3), *WER*, *TTG*, *CPC*, *TRY *and *ETC *are expressed, and owing to diffusion GL3 and EGL3 proteins are also present. This profile corresponds to that reported for cells committed to the non-hair fate (atrichoblasts). The other attractor matches the characteristic profile of cells that will bear root-hairs (trichoblasts), as *GL3*, *EGL3 *and *TTG *are expressed in it and the CPC protein is present.

**Figure 4:**
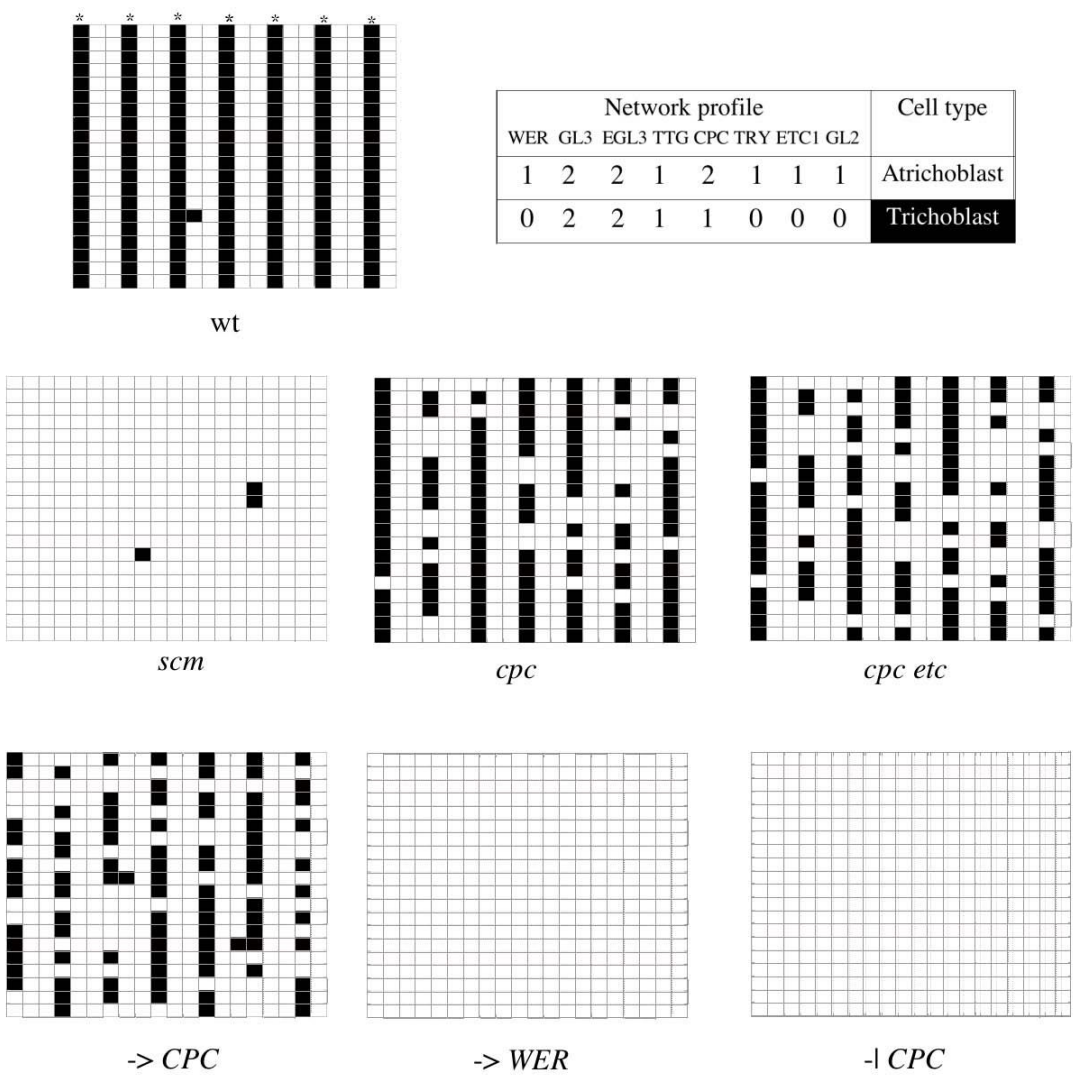
**The model renders cellular patterns similar to those observed in the root epidermis**. Cellular patterns obtained from simulations of wild-type (wt) and mutant networks are consistent with the patterns reported in the literature. Black squares correspond to trichoblasts and white ones to atrichoblasts. Captions under the matrixes indicate the simulated genotype that gave rise to each of them (lower case italics indicate loss of function, -> indicates the simulation of a positive upstream signal, while -| stands for a negative one). Asterisks indicate the hair (H) position. The table shows that the network profiles characteristic of hair and non-hair cells are recovered by the coupled GRN model (B). These simulations were all performed in 20 × 20 matrices with the following parameter values: *D*_*CPC *_= 0.01, *D*_*GL*3 _= 0.01, *D*_*EGL*3 _= 0.01.

**Figure 5:**
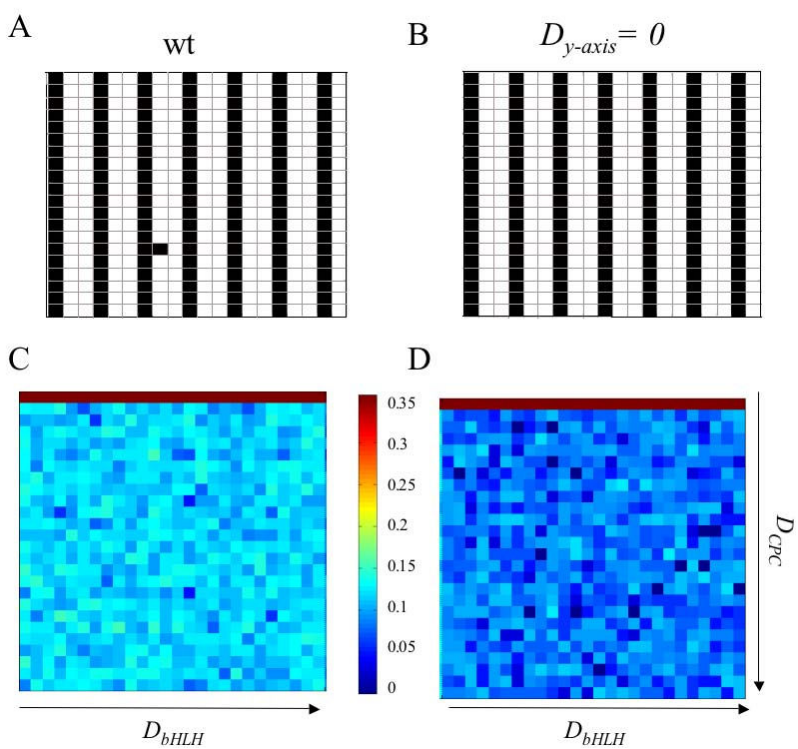
**Spatial root-like pattern is stabilised by differential diffusion in the *x *and *y *axes**. The pattern generated by the coupled GRN model gives rise to a striped pattern with some 'errors' that would correspond to ectopic hairs (A). A similar pattern but with fewer or no errors is obtained when diffusion rate in the *x *axis is larger than that in the *y *axis (*D*_*y*-*axis *_= 0) and the same random seed is taken (B). The parameter spaces for each case are presented below their typical cell arrangements (C), (D). The colour scale indicates the logarithm of the average number of ectopic cell-types for every combination of parameters. Note that, overall, the parameter space obtained for differential diffusion exhibits fewer ectopic trichoblasts.

The simulated spatial cellular pattern of hair and non-hair young cells is very similar to that observed in *Arabidopsis *roots. It is characterised by bands of trichoblasts (black cells in Figure 4) in the H position, where the positional cue is simulated, and bands of atrichoblasts (white cells in Figure 3) in the NH position. This pattern is recovered within a wide range of parameter values (Figure 5). In most of the parameter space generated by varying the diffusion values *D*_*CPC *_and *D*_*bHLH *_from 0 to 0.25 (step size 0.1), the percentage of errors (cells in the NH position without activator complex) is approximately 0.16% (Figure 5). Such positional errors would correspond to ectopic hairs in the root and are smaller than those reported in actual wild-type *Arabidopsis *roots (Col) [41].

The root-like spatial pattern is also robust to different kinds of perturbations. It is resilient to the intensity of the *SCM *signal, measured as a probability value *P*_*s*_, and is also robust to alterations in the output of logical rules: in 20% of the tested changes the number of ectopic cell-types increases, yet the root-like pattern is still clearly discernible. Only 0.06% of the changes modify the nature of attractors or drastically disorganise the spatial arrangement. All simulations shown were performed in a 20 × 20 (rows × columns) lattice. As for the leaf system, variations of the -*A*(*g*(*t*)_[*i*] [*j*]_) term do not affect the wild-type pattern.

Twenty is a fair approximate of the number of epidermal cells in a transversal root section. Given, however, that the pattern may arise in rings of cells, we also tested lattice sizes from 1 × 20 to 40 × 20 and recovered the same results. This suggests that, for a wide range of domain sizes, the root pattern is not sensitive to changes in the number of cells nor to the root section where epidermal cells attain their fate.

### Simulation of mutant GRNs renders spatial patterns that match those observed in actual mutants

In order to validate further the meta-GRN models, we simulated mutations in the GRN and compared the resulting patterns with the reported phenotypes. Mutations were modelled by fixing the state of a node at 0 (loss of function) or 2 (gain of function) throughout the simulations. Overall, the simulated mutants qualitatively resembled those reported in the literature.

We illustrate these simulations with some examples (Figures 3 and 4; [see Additional file 1]) and discuss a few cases in which quantitative differences between simulated and observed patterns were encountered. For the leaf case (Figure 3), the *ttg1 *loss of function simulated mutant gives rise to a homogeneous pattern of no *GL2 *expression that corresponds to a hairless leaf. The simulated *cpc *mutant gives rise to more trichomes than observed in the wild type, but not to more trichome clusters. This is consistent with the available experimental evidence but the increase in hair number that we recover (around 5% more trichomes than in wild type) is smaller that that observed in real *cpc *mutants. As in real plants, the simulated single *etc *mutant does not affect trichome number or distribution, but the *cpc *phenotype is enhanced in the double *cpc etc *simulated mutant. Also compatible with experiments, in the triple *cpc etc try *simulated mutant, the trichome number and clustering probability greatly increase in comparison with the wild-type simulated leaf section. The recovery of a wild-type arrangement in *ttg *simulated mutants overexpressing *GL3 *was also reproduced by our model.

There are two mutant simulations that partially differ from reported leaf phenotypes. The double *cpc etc *simulated mutant gives rise to few clusters, yet not as many as the *try *simulated mutant, while in the actual *cpc etc *mutant trichome clusters are extremely rare. This discrepancy could be because of the fact that in our model all *ETC*s (*ETC1*, 2 and 3) were summarised in one node. On the other hand, simulated overexpression of *GL1 *and *GL3 *genes generates a totally hairy domain that resembles the extremely hairy epidermis of the double overexpression line. A few non-hair cells are still observed, however, in real plants overexpressing *GL1 *and *GL3*. This could be explained if in real plants the overexpression of *GL1 *and/or *GL3 *is not as effective as assumed in the model.

The root meta-GRN patterns of simulated mutants are also very similar to those observed in plants (Figure 4). For instance, the simulated mutant for *cpc *loss of function displays a pattern that matches the increase of ectopic atrichoblasts observed in these mutants. The *scm *simulation gives rise, as in real roots, to a disorganised pattern of trichoblasts and atrichoblasts. The simulated *wer *loss of function shows a hairy phenotype similar to that observed in actual *wer *mutants. Finally, this model also recovers that *etc *enhances *cpc *mutant phenotypes. The leaf and root mutant simulations show that the meta-GRN model is able overall to recover qualitative aspects of the system behaviour and that, in this context, may be helpful for an understanding of pattern formation and providing novel qualitative predictions.

### Novel predictions: the role of cell shape, *GL1*, *GL3 *and *WER *upregulation and GRN interactions with the gibberelic acid pathway and cortex-associated signals

The model we present reproduces documented gene activity configurations in wild-type and mutant plants and also constitutes a powerful predictive tool regarding cellular and genetic mechanisms that may affect the *Arabidopsis *epidermal system.

#### Cell growth and the root hair pattern

In a previous work [18], we postulated that elongation of root epidermal cells could stabilise the early striped pattern by increasing the distance between neighbouring nuclei in the baso-apical direction of the root, thus giving rise to an effective increase of the diffusion rate along this axis. We used the meta-GRN model to simulate a higher diffusion rate on the baso-apical direction than in the radial one. Interestingly, such bias makes the spatial pattern more stable and reduces the average number of ectopic cell-types for the whole parameter space (Figure 5). These new results therefore support the idea that either cell elongation or differential diffusion rates between the longitudinal and radial axes in the root epidermis contribute to stabilising the observed cellular spatial configuration.

#### Trichome patterning and the gibberellin signalling pathway

Density and distribution of root and leaf hair cells change during development and are affected by diverse internal and external signals. Some of these signals are hormones that may affect the GRN modelled here [16,42,43]. We explore the interaction of the leaf GRN and the Gibberelic acid (GA) hormone signalling pathway. Leaf trichome density is positively regulated by GA, among other hormones. Recently, Gan and collaborators [43] found that some transcription factors (*GIS*, *GIS2 *and *ZFP8*) act as GA receptors and act upstream of some elements of the GRN modelled here, biasing cell fate towards trichomes. Indeed, GA receptors upregulate *GL1*, although it is still unknown whether transcription factors like *GIS *act directly on *GL1 *and if such upregulation would suffice to make the leaves hairy.

Several interactions of the GRN could in principle cause *GL1 *upregulation and, at the same time, yield a denser trichome pattern. We simulated GA up and downregulation for all the possible targets within the GRN. We did this by fixing a constant minimum expression of each tested target to 1 (upregulation) or by fixing the node's state to 0 (downregulation) with a probability *P*_*GA *_(equal for all cells) in all cells in the lattice. For simplicity, we assumed that GA is homogeneously distributed in the modelled spatial domain and that the signalling pathway regulates only one of the network's components at a time. We also assume that this hormone does not cause an overexpression effect on its target (i.e. it does not fix the expression state to 2). This last assumption is based on the fact that overexpression of the network components often leads to drastic phenotypes that are not observed in wild-type *Arabidopsis *development and during which GA signalling is present. Results in Table 2 were generated by simulation of two particular *P*_*GA *_values (1 and 0.01), each one representing one of two broad ranges of values that render qualitatively different results.

**Table 2:** Simulation of possible interactions of the GA pathway and the GRN.

	**Trichome number**	**Trichomes adjacent to another one**
wt	19	0
*bHLH*(+)*	**44**	**2**
*bHLH *(+)	400	400
*GL1 *(+)	19	0
*TTG1 *(+)	19	0
*GL2 *(+)	0	0
*GL2 *(+)*	11	0
*CPC *(-)*	19	0
***CPC*(-)**	**20**	**0**
*TRY *(-)	28	8
*ETC *(-)	19	0

Only a positive interaction on *bHLH *genes or a negative one on *CPC *(rows in grey) produces an increase in trichome density and maintains the spaced-out pattern. The sign of interaction is in parentheses. Asterisks indicate an interaction probability *P*_*GA *_< 1, while no asterisk means that this probability was equal to one. The two cases *P*_*GA *_= 1 and *P*_*GA *_< 1 are only shown when their results are different. These simulations were performed in 20 × 20 matrices with the following parameter values: *D*_*CPC *_= 0.05, *D*_*TRR *_= 0.05, *D*_*TTG *_= 0.03.

The simulation results suggest that an increase in *GL3 *and *EGL3 *or a decrease in *TRY *or *CPC *expression leads to a higher density of trichomes and, therefore, to a higher *GL1 *expression levels (Table 2). Both the complete and partial repression of *TRY*, however, yield a higher clustering probability (Table 2). Given that (i) GA partially underlies the differences in trichome density exhibited by certain regions of the plant throughout development, even under normal growth conditions [43,44] and that (ii) clusters are very rare in wild-type plants, it is unlikely that the GA pathway acts directly on *TRY*. In contrast, *bHLH *genes and *CPC *are likely targets of the GA pathway. Although the trichome number increase for *CPC *downregulation is small, it seems that the meta-GRN model underestimates the increase in hair numbers observed in the *cpc *mutant (see previous section in Results) and therefore *CPC *is also a good candidate for GA regulation.

#### Possible cortex-related signals in the root system

We modelled the signal associated with *SCM *as a negative regulation on *WER*, as suggested by recent experimental data [36]. Nevertheless, this may not be the only positional cue biasing the root GRN and it does not affect cell-type patterning in hypocotyls [36], which seems to share the root GRN and also exhibits a striped cell-type arrangement. It is likely that there are additional *SCM*-independent signals and our model may be used to predict the nature of such signals. We made some predictions concerning the possible targets of such signals among GRN nodes.

We simulated several possible targets of both positive and negative putative signals. We thus simulated: upregulation of *WER *in the NH position, upregulation of *CPC *in the H position and downregulation of *CPC *in the NH position. In our model, the negative regulation of *SCM *over *WER *is the one that best recovers the observed root-like striped arrangement (wt in Figure 4). This was the signal used throughout all the simulations mentioned in previous sections, as it is the only one empirically documented. Upregulation of *CPC*, however, also gives rise to a striped pattern (-> *CPC *in Figure 4), although a significantly less stable pattern than the one recovered with the negative signal on *WER*. Negative regulation of *CPC *(-> *CPC *in Figure 4), and upregulation of WER (-> *WER *in Figure 4) render, on the other hand, uniform patterns that are very different from the observed banded ones. Our model therefore suggests that if there were additional signals associated with the root striped cellular patterns, these should act positively on *CPC*.

### *GL3*, *WER *and *GL1 *positive regulation

*GL3 *expression peaks in the cells committed to the trichome identity in leaf epidermis. This suggests that it could be upregulated by the activator complex or by members of such a complex. Yet it was recently shown that its transcriptional regulation is *GL1*-independent [31], although *GL1 *and *TTG1 *seem to regulate the location of the GL3 protein within the cell. Since *CPC *upregulates *GL3 *in the root epidermis, and given that its expression peaks coincide with that of *GL3*, *CPC *could be responsible for *GL3 *activation. We therefore used our model to test whether the simulated trichome pattern remained the same if *CPC *was assumed to activate *GL3*. Our simulations suggest that the upregulation of *GL3 *by *CPC *would cause a drastic increase in trichome density and a clustering probability close to one, contrasting with observed patterns. This result suggests that, if *CPC *upregulated the transcription if *GL3 *in leaf epidermis, another unknown factor would have to restrain *GL3 *expression to trichomes.

As mentioned above, we performed a systematic exploration of the effect of alterations in the logical rules. Interestingly, our simulations suggest that the self-activation of *WER *and *GL1 *is necessary to recover all the wild type and mutant patterns. This prediction is consistent with the fact that these two genes peaks of expression are in the same cells where their inhibitors are also expressed at the highest levels.

## Discussion

We put forward an experimentally-grounded dynamical model that integrates experimental evidence and helps us to study the concerted action of multiple molecular components during *Arabidopsis *hair pattern formation. This constitutes one of the best experimental systems to address questions concerning cell differentiation and spatio-temporal arrangement of cell types. This study uncovered a regulatory module that is sufficient to recover the cell types and spatial configurations characteristic of *Arabidopsis *root and leaf epidermis. The model of coupled GRNs in a cellularised spatial domain was used to provide predictions regarding the effect of unknown interactions, signalling mechanisms acting on the GRNs and cell shape on such epidermal patterning.

Besides the insights of our meta-GRN model concerning the particular cell patterning system under consideration, it makes a general contribution to a fundamental issue in developmental biology: the origin of the information needed for the emergence of cell-type patterns from homogeneous domains. In contrast with single-cell models previously published, we achieved this by coupling single-cell GRNs by explicitly incorporating cell-to-cell communication in a cellularised spatial domain. This enabled us to evaluate whether the observed gene activation profiles are recovered in the meta-GRN.

In the case considered here, the information needed for cell-type arrangement consists of heterogeneous patterns of gene expression and is determined by complex interactions among multiple intra- and extra-cellular factors. Our model therefore recovers the *de novo *formation of positional information from such complex GRNs and suggests that the appearance of this information is very robust. It does not depend on particular initial conditions or domain size, and is resilient in the face of single perturbations in the logical rules, as well as changes in the protein diffusion rates. Robustness has been documented as well for single-cell GRN models [5,6].

As mentioned above, our model also enabled testable predictions on the behaviour of the particular system under study. The first prediction states that cell elongation, or other processes that could alter or bias diffusion rates in the *x *and *y *axes of the root (for example, differential transport rate), have a relevant role in stabilising the spatial pattern generated early in root development. This prediction points at a precise way in which cellular morphological traits may affect cellular patterning and could be tested by crossing a GL2:GUS or GFP marker line plants with altered cell shapes (e.g. [45,46]) and search for alterations in the spatial pattern of this genetic marker of epidermal cell type.

Another prediction concerns the action of GA signalling on the GRN, suggesting that this hormone signalling pathway acts on *CPC *or *bHLH *genes. This prediction is consistent with recent experiments with *bHLH *inducible overexpression lines [47] that suggest that an increase in *bHLH *expression suffices to produce more trichomes. Both *bHLH *and *CPC *actually have GA response motives (PLACE, ), but this does not imply that they respond directly to GA. According to our prediction, mutagenesis of the GA response motives should cause trichome density alterations in response to GA treatments. As data on other signal transduction pathways accumulate, the framework put forward here could be useful for an understanding of how diverse environmental cues are integrated by the regulatory module during epidermal cell-fate determination. This would contribute to a better comprehension of the plastic developmental responses of plant and animals to environmental cues.

The GRN modelled here is sufficient to recover the gene activation profiles and the hair spatial patterns typical of *wt *and mutants' root and leaf epidermis. Our results therefore support the idea that developmental processes, such as cell-type determination and patterning, depend on regulatory modules, which are semi-autonomous with respect to the rest of the genome [48]. According to this and other studies (e.g. [6]) the outputs of these modules (i.e. attractors that correspond to the possible alternative multigene expression profiles) are highly robust in the face of diverse perturbations, suggesting that these may act as patterning modules for a wide range of parameter values in kinetic functions of the network. Moreover, it seems that these modules may have been co-opted during evolution to regulate the decision underlying cell-fate determination of different structures in diverse tissues/organs (e.g. root, hypocotyl and leaf). Indeed, the latter could be the case in plant species with contrasting cellular patterns, in which local traits, such as cell size and shape, positional cues or domain geometry, may affect the type of pattern, even if the regulatory modules have been largely conserved during evolution.

The results presented here also support the idea that the coupled-GRNs system modelled here includes smaller coupled sub-networks that could themselves constitute mechanisms that contribute to the formation or maintenance of cellular patterns. In the leaf system, these mechanisms are: (i) the regulatory and protein-protein interactions between GL3 and TTG1 that give rise to the TTG1-trapping/depletion mechanism postulated by Bouyer and collaborators [28], (ii) the so-called activator-inhibitor system conformed by the activators of GL2 and its inhibitors [17,18], and (iii) the feedback loop established between TRY and GL2 [29,30]. Although these sub-networks or motifs could be important or even sufficient for the generation and maintenance of the epidermal cellular patterns, independently of the rest of the GRN, it is likely that they all act redundantly in the generation of such patterns in a robust manner. The latter seems to be the case as, for instance, our results suggest that TTG1 diffusion is not necessary for patterning when the whole GRN is present. Experimental data also show that wild-type cell configurations are observed in *ttg1 *mutants when *GL3 *is overexpressed [40]. Since these mechanisms are not independent of each other, the meta-GRN model encompassing them all in conjunction with cell-cell communication is useful for studying the spatio-temporal patterning. The interlinkage of redundant sub-networks found in this system supports the hypothesis that interconnection among feed-back loops or small nonlinear systems confers robustness to biological networks and may constitute an important trait in network evolution [49].

## Conclusion

The model and results presented enable us to conclude that cell-type determination and patterning in leaf and root epidermis of *Arabidopsis *are regulated by a complex GRN that encompasses redundant sub-networks. Since all these sub-networks involve cell-to-cell communication, the spatial meta-GRN model is central to studying how these interact with each other, giving rise to the information needed for cellular patterning. Unlike the importance of gene redundancy, the relevance and implications of sub-network redundancy remain largely unexplored. This work is one of the first contributions regarding this issue.

We could also conclude that the meta-GRN qualitatively reproduces pattern formation in the system under study. This is why this model could be used to state precise predictions, namely, that the GA pathway acts on *CPC *or the *bHLH *genes, that *GL1 *and *WER *are self-upregulated, that root cell shape has a stabilizing role on the banded pattern and that new cortex-related biasing signals may upregulate *CPC*.

Future developments of the meta-GRN model proposed here should allow for cell proliferation or continuous gradients in pattern formation. Nevertheless, the model presented here constitutes a starting-point from which to integrate future experimental evidence of the intracellular GRNs themselves (e.g. epigenetic regulation), as well as diverse cellular processes. As further meta-GRN models are postulated and validated for diverse biological systems, we will be able to evaluate which types of intracellular GRNs, regulatory motifs or sub-networks, as well as coupling mechanisms, underlie patterning and morphogenesis in living organisms.

As exemplified by the GRN studied here, development depends upon non-additive interactions occurring in a wide range of spatial and temporal scales. Accomplishing the paramount task of understanding how robust patterns arise during development may therefore be facilitated by computational or mathematical models that bridge the divide between dynamics of interactions at the microscopic level and the origin and evolution of morphological qualities. Indeed, models like the one put forward here constitute useful tools for integrating experimental work, hinting at new experiments and providing novel insights into the complex interactions that underlie patterning processes and, therefore, into developmental and evolutionary biology.

## Authors' contributions

MB participated in the study design, specified the GRN model, wrote the computer programs and carried out the simulations, participated in the discussion of results and contributed to writing the manuscript. CES participated in the study and computer program design, as well as in the discussion of results. PPL participated in the study design, the mathematical formulation of the model, and in the discussion of results. EAB established the project and coordinated this study, participated in the study design, in the analysis of results and in writing the manuscript. All authors read and approved the final manuscript.

## Note added in proof

In the root meta-GRN model we assume that *WER *activates itself. This interaction is not essential for the formation of wild-type pattern, but it is required for the formation of the pattern observed in the absence of *SCM*. While this paper was under review, Savage and collaborators (2008) [50] put forward a mathematical model and a patterning mechanism that do not involve *WER*'s self-activation. These authors also provide new data regarding *WER *regulation that are consistent with their proposal. However, the data does not reject the possibility that *WER *self-activates, as they are also consistent, for instance, with the combined activity of a self-activating loop and another input activating *WER*. According to available empirical data, both the model presented here and that proposed by Savage and collaborators (2008) [50] appear to be plausible. Further tests could help find if a mechanism involving *WER*'s self-activation, another mechanism, or the coupled activity of more than one mechanism are important for cell-type arrangement in *Arabidopsis *root epidermis. Interestingly, the existence of more than one patterning mechanisms would emphasize the importance of sub-network redundancy in developmental networks.

## Supplementary Material

Additional file 1 **This file contains the thorough topology and updating GRN functions.** Click here for file
