# Supplementary material for: Interlinked nonlinear subnetworks underlie the formation of robust cellular patterns in Arabidopsis epidermis: a dynamic spatial model
Source: BMC Syst Biol. 2008 Nov 17;2:98. doi: 10.1186/1752-0509-2-98 (PMC2600786; doi:10.1186/1752-0509-2-98)
Supplement: Additional file 1 — This file contains the thorough topology and updating GRN functions. [file 1752-0509-2-98-S1.doc]

**Additional material for** ***Interlinked nonlinear sub-networks underlie the formation of cellular patterns in* Arabidopsis *epidermis: a spatial model of coupled gene regulatory networks.***

Mariana Benítez, Carlos Espinosa-Soto1, Pablo Padilla-Longoria and Elena R. Alvarez-Buylla[[1]](#footnote-2)

Logical rules constitute the precise description of the network topology and of the function with which the nodes’ values are calculated in every time-step:

*g_n_(t+1)=F_n_(g_n1_(t),g_n2_(t),..., g_nk_(t)).*

Values of the regulators are on the left side of the tables, while the regulated node or nodes are on the right side. X indicates that, given the entrances of the rest of the table, the node can take any value. Y and Z are temporal variables that integrate the values of two groups of nodes (*WER/GL1, GL3, EGL3, TTG1* and *TRY, CPC, ETC,* respectively) and are then used to estimate the expression value of other nodes. The definition of Y and Z facilitates the presentation and implementation of the logical rules, but does not affect the synchronic updating schema or the interactions among elements of the network.

1. Logical rules for updating the discrete gene regulatory network model for the leaf epidermis.

| Inputs | | | | Output |
| --- | --- | --- | --- | --- |
| GL1 | GL3 | EGL3 | TTG1 | Y |
| 0 | X | X | X | 0 |
| X | GL3+EGL3=0 | | X | 0 |
| X | GL3+EGL3<3 | | 0 | 0 |
| 1 | 2 | 2 | 1,2 | 2 |
| 2 | 2 | X | 1,2 | 2 |
| 2 | 1 | 2 | 1,2 | 2 |
| E L S E | | | | 1 |

| Inputs | | | Output |
| --- | --- | --- | --- |
| TRY | CPC | ETC | Z |
| 0 | CPC+ETC<2 | | 0 |
| 0 | 2 | 0 | 0 |
| 2 | CPC+ETC>0 | | 2 |
| 1 | 2 | X | 2 |
| E L S E | | | 1 |

| Inputs | | Outputs | | | | | | |
| --- | --- | --- | --- | --- | --- | --- | --- | --- |
| Y | Z | GL1 | GL3 | EGL3 | GL2 | TTG1 | ETC | CPC |
| 0 | 0 | 1 | 0 | 0 | 0 | 1 | 0 | 0 |
| Y<Z | | 1 | 0 | 0 | 0 | 1 | 0 | 0 |
| 1 | 0 | 2 | 1 | 1 | 1 | 1 | 1 | 1 |
| 2 | 0 | 2 | 1 | 1 | 2 | 1 | 2 | 2 |
| 1 | 1 | 2 | 1 | 1 | 1 | 1 | 1 | 1 |
| 2 | 1,2 | 2 | 1 | 1 | 2 | 1 | 2 | 2 |

| Input | Output |
| --- | --- |
| GL2 | TRY |
| 0 | 0 |
| 1 | 1 |
| 2 | 2 |

1. Logical rules for updating the discrete gene regulatory network model for the root epidermis.

| Inputs | | | | Output |
| --- | --- | --- | --- | --- |
| WER | GL3 | EGL3 | TTG1 | Y |
| 0 | X | X | X | 0 |
| X | GL3+EGL3<3 | | 0 | 0 |
| X | 0 | 0 | 1 | 0 |
| 1,2 | 2 | 2 | X | 2 |
| 2 | 1 | 2 | 1 | 2 |
| 2 | 2 | 0,1 | 1 | 2 |
| E L S E | | | | 1 |

| Inputs | | | Output |
| --- | --- | --- | --- |
| CPC | TRY | ETC | Z |
| 0 | 0,1 | 0 | 0 |
| X | 2 | X | 2 |
| 2 | X | X | 2 |
| E L S E | | | 1 |

| Inputs | | Outputs | | | | | | |
| --- | --- | --- | --- | --- | --- | --- | --- | --- |
| Y | Z | GL3 | EGL3 | CPC | WER | TTG1 | GL2 | ETC |
| 0 | 0 | 1 | 1 | 0 | 1 | 1 | 0 | 0 |
| 0 | 1 | 2 | 2 | 0 | 0 | 1 | 0 | 0 |
| 0 | 2 | 2 | 2 | 0 | 0 | 1 | 0 | 0 |
| 1 | 0 | 0 | 0 | 1 | 1 | 1 | 1 | 1 |
| 1 | 1 | 1 | 1 | 0 | 0 | 1 | 0 | 0 |
| 1 | 2 | 1 | 1 | 0 | 0 | 1 | 0 | 0 |
| 2 | 0 | 0 | 0 | 2 | 2 | 1 | 2 | 1 |
| 2 | 1 | 0 | 0 | 2 | 2 | 1 | 2 | 1 |
| 2 | 2 | 2 | 2 | 1 | 1 | 1 | 1 | 1 |

| Input | Output |
| --- | --- |
| GL2 | TRY |
| 0 | 0 |
| 1 | 1 |
| 2 | 2 |

**TABLE S1.** Reported and simulated phenotypes corresponding to single and double loss of function (italics low case), as well as to overexpression (+) lines for the leaf meta-GRN. All simulations were carried out with the same parameter values (*D_CPC_= 0.05, D_TRR_= 0.05, D_TTG_= 0.03*).

| Cell type patterns in the leaf epidermis of Arabidopsis thaliana | | |
| --- | --- | --- |
| Genotype | Reported phenotype | Simulated phenotype |
| Wild type | Trichomes spaced out with no clusters | Trichomes spaced out with no clusters |
| *cpc* | More trichomes than in wt, no clusters | ~5% more tricomes than in wt, no clusters |
| *try* | High cluster probability | High cluster probability |
| *etc* | No phenotype | No phenotype |
| cpc etc | Enhancement of the *cpc* phenotype | Enhancement of the *cpc* phenotype with few clusters |
| *cpc etc try* | High number of trichomes and clusters | High number of trichomes and clusters |
| *cpc try* | High cluster probability (*try*-like phenotype) | High cluster probability (*try*-like phenotype) |
| *ttg* | No trichomes | No trichomes |
| *bhlh* | No trichomes | No trichomes |
| *bHLH* (+) | More trichomes than in wt | More trichomes than in wt |
| *ttg* *bHLH* (+) | Wt phenotype | Wt phenotype |
| *ttg GL1(+)* | *ttg* phenotype | *ttg* phenotype |
| *gl1* | No trichomes | No trichomes |
| *GL1 (+) bHLH (+)* | Many more trichomes than in wt, but still with few pavemente cells | Only trichomes and no pavement cells |

**TABLE S2.** Reported and simulated phenotypes corresponding to single and double loss of function (italics low case), as well as to overexpression (+) lines for the root meta-GRN. All simulations were carried out with the same parameter values (*D_CPC_= 0.01, D_GL3_= 0.01, D_EGL3_= 0.01*).

| Cell type patterns in the root epidermis of Arabidopsis thaliana | | |
| --- | --- | --- |
| Genotype | Reported phenotype | Simulated phenotype |
| Wild type | Alternated bands of hair and no-hair cells | Alternated bands of hair and no-hair cells |
| *scm* | Hairs and no-hair cells without banded pattern. | Hairs and no-hair cells without banded pattern. |
| *wer* | Many ectopic hairs | Only hair cells |
| *bhlh* | Remarkable increase in the number of hair cells | Remarkable increase in the number of hair cells |
| *cpc* | Ectopic atrichoblasts | Ectopic atrichoblasts |
| *try* | Ectopic atrichoblasts | Ectopic atrichoblasts |
| *cpc etc* | Enhanced *cpc* phenotype | Enhanced *cpc* phenotype |
| CPC (+) | Ectopic hair cells | Ectopic hair cells |
| WER (+) | Wt phenotype | Wt-like phenotype with some ectopic cells |

1. # eabuylla@gmail.com

   [↑](#footnote-ref-2)
